# Reciprocity

**Published:** 1898-12

**Authors:** 


ARTICLE VII.

### RECIPROCITY.

There are over fifty institutions in the United States engaged in the teaching of dentistry. At the last meeting of the National Association of Dental Faculties, thirty-eight of these institutions were members of the association, and the applications of the others for membership were in various stages of progress. We may soon hope that all the various American dental schools will own allegiance to the National Association, which has already done so much towards leveling up the educational standard of dentistry in the United States. A letter was read at the same meeting from Dr. Wm. Mitchell, of London, who, as the mouthpiece of the American Dental Club of London, complained that the degrees of the colleges subscribing to the association were not recognized in Europe. This they think is a great injustice, both to the colleges and to the graduates, past and future.

The attempt to standardize curricula and examinations is coming more and more to the front. Formerly, and still to a great extent, each school, college or university set its own curriculum and examination and appointed its own examiners. Now the attempt is being made more or less successfully to centralize and standardize all such educational machinery, and so avoid the anomaly of very different grades of medical and surgical training endowing the respective possessors with the same privileges. The General Medical Council is doing for dentistry in [these islands what the National Association is doing for the States. But before reciprocity between two countries can be arranged, the same process must be carried on between the countries as is already being conducted in the various colleges in the individual countries. For instance, before reciprocity can become an accomplished fact in dental matters between the States and Ourselves, the educational standard of both must be brought on a par. Without being carping or vain-glorious, we are compelled to affirm that dental education in the States is open to criticisms which do not apply to us. Colleges or universities among our cousins are often "run" as purely private money-making concerns. Inducements are in some cases held out as to the ease with which the curriculum can be gone through, and statements made as to the rare occurrence of failure in the final examinations. These statements, unfortunately, are often only too true, as the teachers are the examiners and have a financial interest in passing as many candidates as possible. We have reason too to suppose that the "beneficiary" or "scholarship" student system has been abused, as has also the system of bestowing honorary dental qualifications on more or less worthy objects. The eagerness of the competing colleges is also borne out by the fact that students have completed their curriculum, while still owing their fees, a thing unknown among us, where fees are always paid in advance.

We do not think that a joint committee of British and American dental educationists will be formed in the near future, but we do think that a request formulated by the American Dental Club is worthy of all attention. This request is that authorities should institute inquiries so that each country may know what credit ought to be given educational certificates from institutions in other lands. We do not permit American graduates to register here, simply because their curriculum differs from ours; but where a certificate from a high-class American university is presented to our authorities, the candidate is given full credit for that portion of it which corresponds to the similar portion of our curriculum. In the same way a graduate of our colleges is given credit for his work already done when he wishes to graduate at an American university. This is as far as reciprocity has gone at present, and it seems to us no greater "injustice" one way than the other, except perhaps that, as the numerous American colleges have been turning out graduates in a very much shorter period than is the case with us, there is a larger number of them looking for spheres of work abroad. The tendency with our colleges, which are very little influenced by financial considerations, is to make our curriculum more severe, from the preliminary entrance examination onwards. The examiners are not teachers in our dental schools, and are unbiased in their work of testing the knowledge of candidates. The consequence is that the ordeal for the L. D. S. diploma is a severe one, and is still becoming more searching. When the American schools can meet us on level ground it will be time enough to discuss reciprocity, and meanwhile we welcome the efforts of an honorable body such as the National Association, which, working for the advancement of the profession, is assisting in the progress and welfare of mankind.—*British Journal of Dental Science.*

